# Evaluating emergency response at a hospital near the Gaza border within 24 h of increased conflict

**DOI:** 10.1186/s12873-024-00964-5

**Published:** 2024-03-21

**Authors:** Hezi Levi, Gili Givaty, Yaniv S. Ovadia, Yaniv Alon, Mor Saban

**Affiliations:** 1 Management, Barzilai University Medical Center, 7830604 Ashkelon, Israel; 2 Research and Development Authority, Barzilai University Medical Center, 7830604 Ashkelon, Israel; 3 Nursing Department, School of Health Professions, Faculty of Medical and Health Sciences, Tel Aviv University, Tel Aviv, 69978 Israel

**Keywords:** Emergency medicine, Mass casualty, Trauma care, Frontline hospitals, Armed conflict, Healthcare delivery

## Abstract

**Background:**

Frontline hospitals near active hostilities face unique challenges in delivering emergency care amid threats to infrastructure and personnel safety. Existing literature focuses on individual aspects like mass casualty protocols or medical neutrality, with limited analysis of operating acute services directly under fire.

**Objectives:**

To describe the experience of a hospital situated meters from hostilities and analyze strategies implemented for triage, expanding surge capacity, and maintaining continuity of care during attacks with limited medical staff availability due to hazardous conditions. A focus will be placed on assessing how the hospital functioned and adapted care delivery models in the event of staffing limitations preventing all teams from arriving on site.

**Methods:**

A retrospective case study was conducted of patient records from Barzilai University Medical Center at Ashkelon (BUMCA) Medical Center in Israel within the first 24 h after escalated conflict began on October 7, 2023. Data on 232 admissions were analyzed regarding demographics, treatment protocols, time to disposition, and mortality. Missile alert data correlated patient surges to attacks. Statistical and geospatial analyses were performed.

**Results:**

Patients predominantly male soldiers exhibited blast/multisystem trauma. Patient surges at the hospital were found to be correlated with the detection of incoming missile attacks from Gaza within 60 min of launch. While 131 (56%) patients were discharged and 55 (24%) transferred within 24 h, probabilities of survival declined over time reflecting injury severity limitations. 31 deaths occurred from severe presentation.

**Conclusion:**

Insights gleaned provide a compelling case study on managing mass casualties at the true frontlines. By disseminating BUMCA's trauma response experience, strategies can strengthen frontline hospital protocols optimizing emergency care delivery during hazardous armed conflicts through dynamic surge capacity expansion, early intervention prioritization, and infrastructure/personnel protection measures informed by risks.

## Introduction

During instances of armed conflicts, frontline civil hospitals situated in proximity to active hostilities encounter formidable obstacles in effectively delivering emergency care while also ensuring the protection of infrastructure and personnel amidst surrounding dangers [1–3]. Several reports have documented the toll on hospitals struggling to treat mass casualty surges during conflicts. However, few studies examine maintaining healthcare delivery from the perspective of a facility confronting threats to staff, patients and infrastructure, while delivering frontline care under fire. Existing guidelines address domains like mass casualty event protocols and personnel protection amid crises; however limited research analyzes operating acute services at the true frontlines of conflict [4–6].

**Fig. 1:**
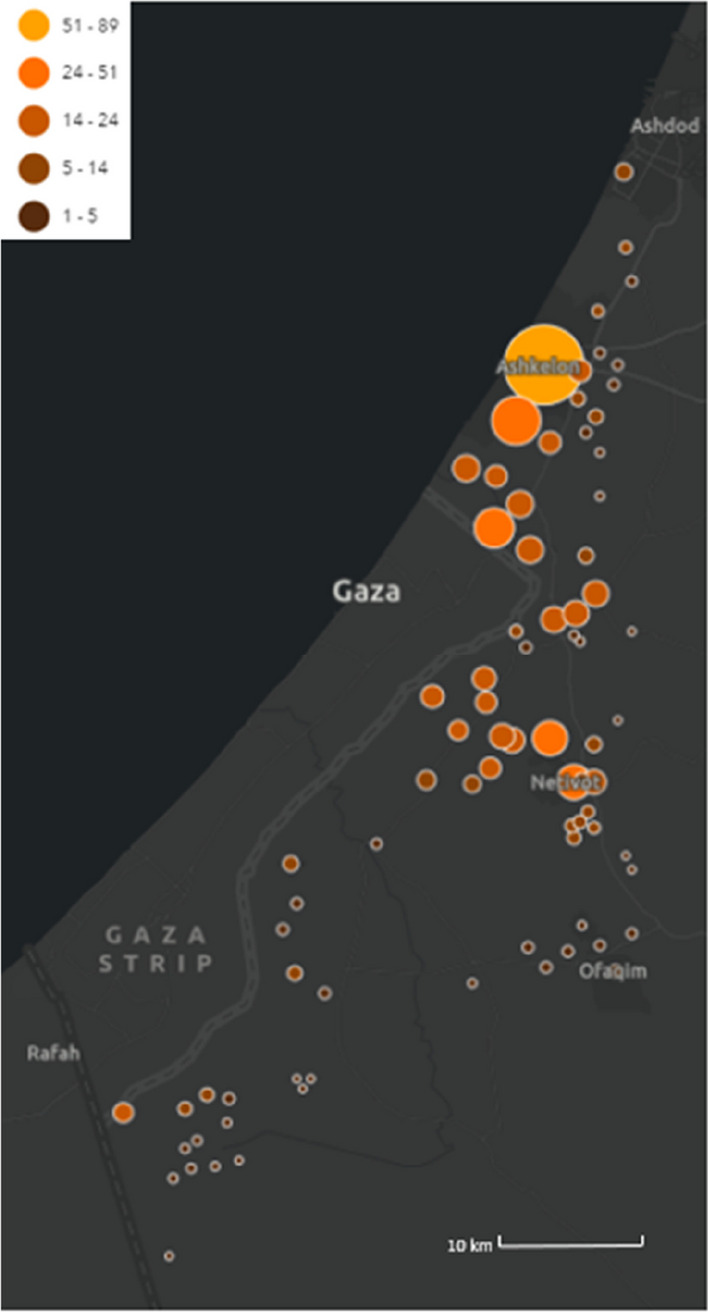
Map depicting missile alerts during the initial 24 h of the Hamas-Israel war. ^a^ The presented data pertains to the region encompassing BUMCA, situated in Ashkelon, where Hamas missile targets were more concentrated compared to the surrounding areas. It is crucial to note that the data points are solely updated in response to missile alerts received through the Home Front Command website. Consequently, they do not accurately reflect the actual count of missiles launched, which is higher than what is represented in the provided dataset ^a^Armed conflict which began on October 7th 2023, when Hamas terrorist from Gaza attacked Israel with rockets, invaded into Israel and brutally murdered thousands of civilians (Jewish, Christian and Muslim) among other violent acts. BUMCA = Barzilai University Medical Centre, Ashkelon, Israel

The experience of Barzilai University Medical Center at Ashkelon city, Israel (BUMCA), located 19.9 km from the border of the Gaza Strip (Fig. 1), offers insights into challenges faced by frontline hospitals [7]. As one of the hospitals within the conflict zone, BUMCA found itself at the center of the crisis with daily rockets targeting surrounding neighborhoods and directly into the hospital itself [7].

When facing threats that could increase patient volumes, the healthcare organization's leadership and clinicians had to develop strategies to effectively manage surging numbers of casualties while also protecting staff, patients, and ongoing care delivery. Real-world solutions implemented by the organization for key tasks like triage, expanding surge capacity, and maintaining continuity of services during emergencies will be described. Specifically, their approaches relating to rapid triage protocols, flexible expansion of treatment spaces and staffing models, and use of telehealth to ensure access to care when on-site presence was limited, will be detailed. Providing this overview helps readers understand what practical solutions and experiences from the organization's emergency response efforts will be explored. Their coordinated actions for addressing mass casualty surges while sustaining normal operations may offer valuable lessons for other healthcare organizations in similar circumstances [7–9].

A review of literature on trauma care and management of mass casualty incidents offers relevant context. Franke et al. described the standard of care for traumatic injuries commonly caused by explosive devices and firearms from the pre-hospital setting through to emergency departments. [10] The authors emphasized the importance of proper triage, early hemorrhage control through techniques such as tourniquet application and rapid surgical intervention to optimize outcomes for critically injured patients [10]. Additional studies have analyzed trauma management strategies for patients suffering from blast injuries or multiple trauma [11].

Early diagnosis through imaging modalities, timely hemorrhage control and surgical intervention were shown to be vital for saving lives and limbs, especially in environments with limited resources [12,13]. Together, these studies emphasize the need for well-executed damage control resuscitation and surgery protocols to effectively treat large volumes of severely injured patients, as medical facilities like BUMCA often encounter during conflicts.

Therefore, our objective were (a) to describe the clinical management from BUMCA's experience in the initial 24 h after the escalation of armed conflict began which began on October 7th 2023, when Members of Hamas attacked Israeli communities with rockets meanwhile invading Israel and murdering over a thousand civilians (Jewish, Christian, and Muslim) initiating the Hamas-Israel war (b) to investigate BUMCA's response to the surge of trauma patients admitted during this time period and (c) to glean practical strategies that could help other hospitals facing similar fronts better plan for efficiently delivering frontline emergency care under threatening conditions.

## Methods

### Settings and time frame

This retrospective study analyzed anonymized data from Inpatient records admitted to the emergency department of BUMCA within the first 24 h of Hamas-Israel war, that took place between October 7 (6:50 AM)- October 8 (5:44 AM), 2023 (the time frame).

### Data collection

Data were collected for patients received at BUMCA Medical Center during the initial 24-h period following the escalation of conflict on October 7, 2023. Anonymized patient data elements gathered included: initial triage category upon arrival to the Emergency Department (ED), ED receiving area (critical care resuscitation bay, stable condition treatment room, minor conditions area), admission date, military affiliation, age, gender, type of arrival (ambulance, walk-in, etc.), time of arrival, and vital status at hospital discharge. The ED receiving area referred to the location patients were first brought for initial assessment and stabilization within the ED, including designated spaces for those requiring critical care, stable condition monitoring, or minor treatment. Missile alert data for the same 24-h period were obtained from the public website of the Israel Home Front Command (https://www.oref.org.il/en) [14].

Alerts document the timing and location of incoming rocket and missile attacks. This information was extracted, recorded and later analyzed as part of the study. Times of missile impacts were correlated with emergency department patient surge volumes and acuity levels.

### Statistical analysis

Statistical analyses were performed using Python version 3.10. The ‘chi2_contingency’ function from the scikit-learn library (version 1.1.1) was employed for *X*^[[2]]^ tests on categorical variables. Additionally, t-tests for quantitative variables were conducted using the ‘ttest_ind’ function from the SciPy library (Version: 1.10.1). The outcomes of these tests are presented in Table 1.

**Table 1:** Clinical characteristics of patients admitted to the BUMCA's ED during the first 24 h of the Hamas-Israel war (*n* = 232)^a^

	**Children**	**Eyes**	**Gynecology**	**Internal medicine**	**Non-ambulatory**	**Mental health**	**Orthopedics**	**Specialized unit for high risk pregnancy**	**Surgery**	**Trauma Center**	**Urology**	**Total**	***P value***
n (%)	13 (5.6)	1 (0.43)	1 (0.43)	15 (6.46)	92 (40)	26 (11.21)	21 (9.05)	1 (0.43)	45 (19.4)	15 (6.47)	2 (0.86)	**232 (100)**	-
Gender													
Male (%)	9 (69.23)	1 (100)	0 (0)	6 (2.58)	69 (75)	11 (42.3)	14 (66.7)	0 (0)	30 (66.67)	13 (86.67)	2 (100)	**155 (66.8)**	0.29
Female (%)	4(30.76)	0 (0)	1 (100)	9 (3.87)	23 (25)	15 (57.69)	7 (33.33)	1 (100)	15 (33.33)	2 (13.3)	0 (0)	**77 (33.2)**	
Age (y)	7.08 ± 5.31	37 ± 0	40 ± 0	52.9 ± 11.4	34.6 ± 16.4	39.23 ± 18.5	35.52 ± 15.63	32 ± 0	43.8 ± 20.5	51.87 ± 38.8	46 ± 28.28	**38.2 ± 18.19**	< 0.001
Movement type													
Deceased n (%)	0 (0)	0 (0)	0 (0)	0 (0)	31 (33.7)	0 (0)	0 (0)	0 (0)	0 (0)	4 (26.67)	0 (0)	**35 (15.1)**	< 0.001
Transition from the emergency room to hospitalization n (%)	0 (0)	0 (0)	0 (0)	2 (0)	0 (0)	0 (0)	0 (0)	0 (0)	1 (2.22)	0 (0)	0 (0)	**3 (1.29)**	
Released home (%)	12 (92.31)	1 (100%)	1	8 (70)	35 (38.04)	24 (92.31)	14 (66.67)	1 (100%)	30 (66.67)	4 (26.67)	2 (100)	**132 (56.9)**	
Temporary stay in the emergency room n (%)	0 (0)	0 (0)	0 (0)	0 (0)	0 (0)	1 (3.85)	0 (0)	0 (0)	0 (0)	0 (0)	0 (0)	**1 (0.43)**	
Independent departure n (%)	0 (0)	0 (0)	0 (0)	0 (0)	1	1 (3.85)	1 (4.76)	0 (0)	1 (2.22)	1 (6.67)	0 (0)	**5 (2.16)**	
Relocation from the medical department n (%)	0 (0)	0 (0)	0 (0)	0 (0)	0 (0)	0 (0)	0 (0)	0 (0)	1 (2.22)	0 (0)	0 (0)	**1 (0.43)**	
Refusal to receive treatment n (%)	0 (0)	0 (0)	0 (0)	0 (0)	0 (0)	0 (0)	1 (4.76)	0 (0)	0 (0)	0 (0)	0 (0)	**1 (0.43)**	
Transfer to another institution n (%)	1 (7.7)	0 (0)	0 (0)	3 (30)	25 (27.17)	0 (0)	5 (23.81)	0 (0)	12 (26.67)	6 (40)	0 (0)	**52 (22.4)**	

*BUMCA* Barzilai Unversity Medical Center, Ashkelon, Israel, *ED* emergency department

The table is structured into various patient groups describing their characteristics based on demographics and injury or illnesses sustained. This including Children, Non-ambulatory, Internal medicine, Surgery, Trauma center, Orthopedics, Mental health, Urology, Gynecology, Specialized unit for high risk pregnancy, and Eyes. Each patient group is further categorized based on movement type, indicating the destinations of patients following their initial screening upon arrival at the emergency room. The movement types encompass: released home, transfer to another institution, transition from the emergency room to hospitalization, independent departure, deceased, relocation from the medical department, refusal to receive treatment, and temporary stay in the emergency room. Statistical analyses were conducted to assess the significance of observed differences. *P*-values were computed employing t-tests for quantitative variables and chi-square tests for categorical variables, facilitating a comprehensive evaluation of the diverse movement patterns within and across patient groups

± are means ± SD

^a^Armed conflict which began on October 7th 2023, when Hamas terrorist from Gaza attacked Israel with rockets, invaded into Israel and brutally murdered thousands of civilians (Jewish, Christian and Muslim) among other violant acts

#### Geographic visualization

Power BI (Version: 2.122.746.0 64-bit, October 2023) was utilized to create illustrations map, integrating with the ArcGIS platform for enhanced mapping capabilities and visualization.

#### Temporal visualization

Temporal trends in the number of patients and missile impacts were visualized using the Plotly library (Version: 5.15.0). Line plots with dual y-axes were generated to illustrate the correlation between patient admissions and missile impacts over time.

#### Survival analysis

Survival analysis was conducted using the Lifelines library (Version: 0.27.7). The Kaplan–Meier plot was created to depict the probability of survival over time for patients arriving at the emergency department (ED) during the specified period.

## Result

Within the first 24 h of the conflict, BUMCA's ED received 232 patients from areas near Hamas strikes. Fig. 1 depicts a map showing missile alerts during the initial 24 h of the Hamas-Israel war. The map pertains to the region surrounding BUMCA in Ashkelon, where Hamas missile targets were more concentrated compared to surrounding areas.

Table 1 shows the clinical characteristics of patients admitted to BUMCA's ER, including demographic data, mechanism of injury, and referral sources. Out of 232 total ED patients, 155 were male patients comprising 66.8% of the cohort.

Of the 232 casualties, 155 (66.8%) were admitted for inpatient care. The majority (*n* = 95, 61.3%) of admitted patients were stabilized in the ED resuscitation bay prior to ward transfer. The remaining admitted patients (*n* = 60, 38.7%) were deemed too clinically unstable for ward care upon initial assessment due to cardiovascular compromise, altered mental status, or need for invasive monitoring/support. These high acuity non-ambulatory patients were directly admitted to the ICUs if beds were available. Overall, 31 of the 34 deceased patients hospital-wide (88%) were initially admitted to the non-ambulatory ward, highlighting the particularly high acuity and mortality of this subset of casualties from the mass casualty event. Table 1 also shows that 56% of patients (*n* = 131) were discharged from hospital treatment within 24 h and 23% (*n* = 55) were transferred to another institution for treatments beyond the hospital's capacity, all within the 24-h timeframe.

**Fig. 2:**
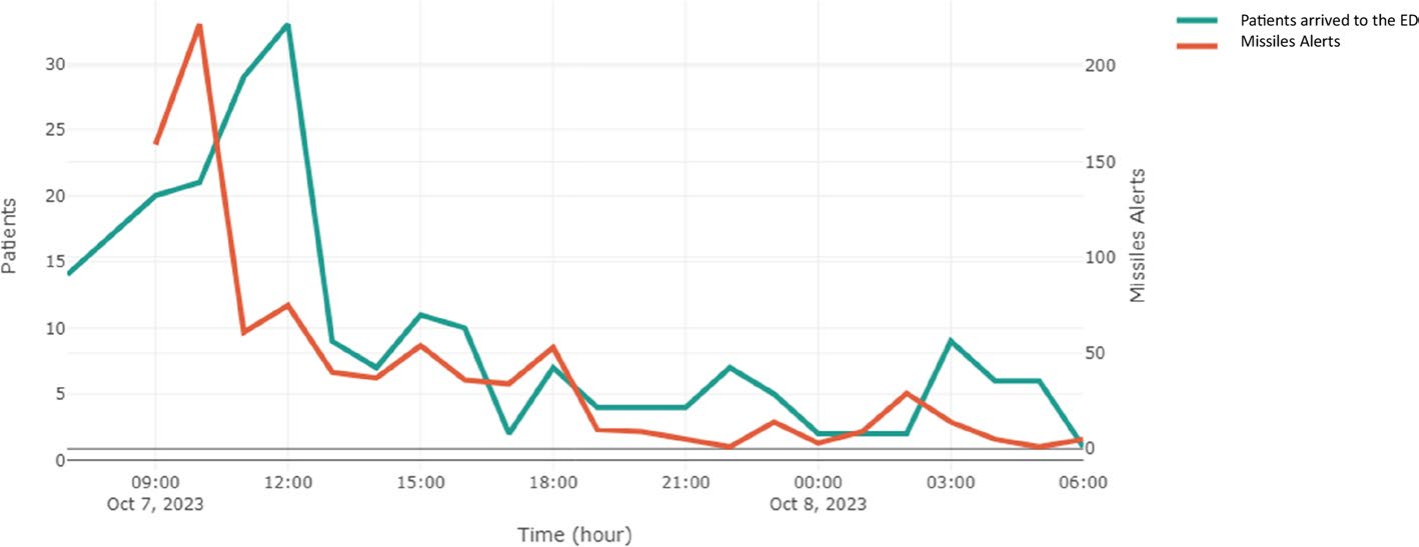
Number of patients arrived at the BUMCA's ER amid missile attacks launched from Gaza toward southern Israel, within the initial 24 h of the Hamas-Israel war. ^a^ The graph illustrates the correlation between the number of patients (left y-axis) and the number of missile alerts (right y-axis) on an hourly basis (x-axis). The absence of data at the beginning of the missile alerts plot is attributed to a data overload, a consequence of the simultaneous firing of thousands of missiles within the initial 3 h ^a^Armed conflict which began on October 7th 2023, when Hamas terrorist from Gaza attacked Israel with rockets, invaded into Israel and brutally murdered thousands of civilians (Jewish, Christian and Muslim) among other violant acts. BUMCA = Barzilai Unversity Medical Center, Ashkelon, Israel; ED = emergency department

**Fig. 3:**
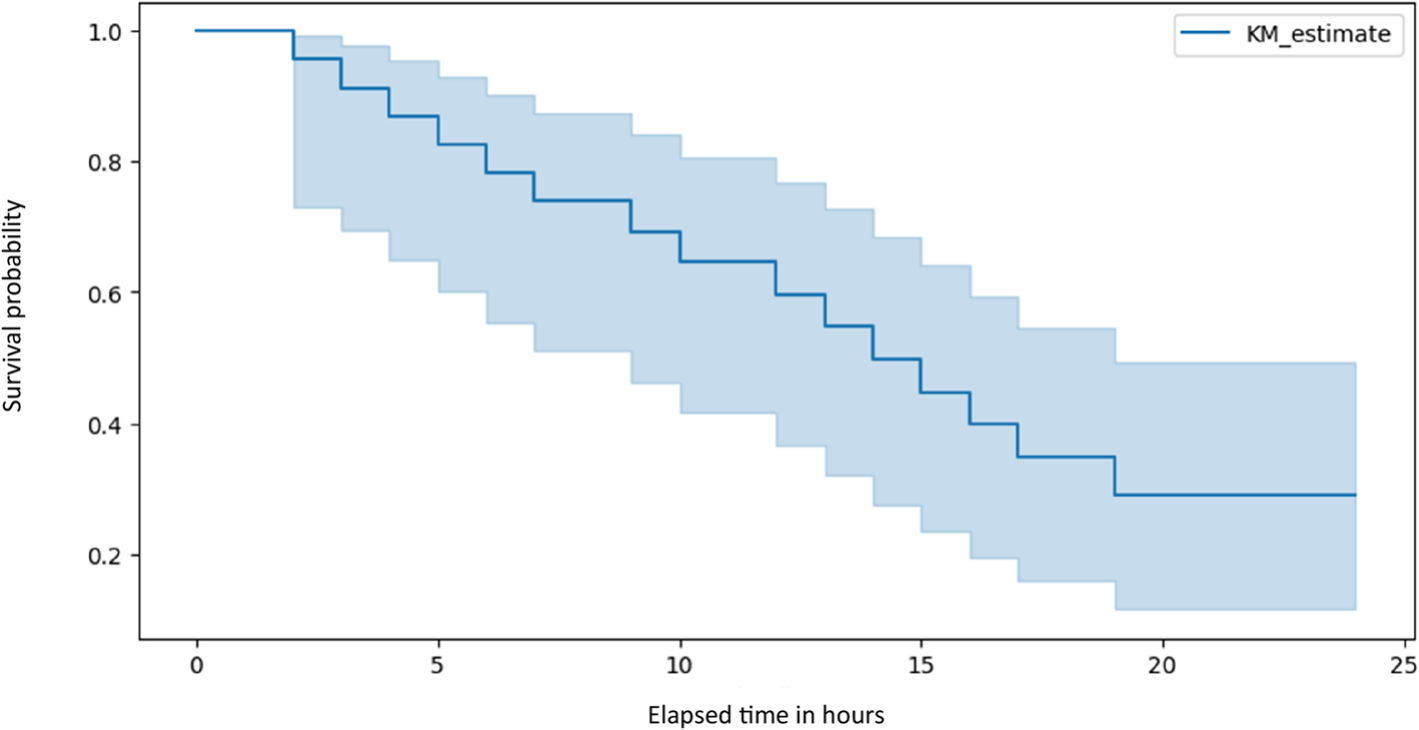
Kaplan–Meier plot depicting patients who presented at BUMCA's ER within the initial 24 h of the Hamas-Israel war. ^a^ The Kaplan–Meier plot for patients arrived at the ER, shows the probability of survival over time ^b^ for these patients: the estimated survival probability (y-axis) at each time point (x-axis) ^a^Armed conflict which began on October 7th 2023, when Hamas terrorist from Gaza attacked Israel with rockets, invaded into Israel and brutally murdered thousands of civilians (Jewish, Christian and Muslim) among other violant acts. ^b^1.0 = 100%. BUMCA = Barzilai Unversity Medical Center, Ashkelon, Israel; ER = emergency room

The Hamas missile attacks on Israel and the influx of patients to the ED exhibit a correlated pattern, as illustrated in Fig. 2, with a lag of 30–60 min between them. This implies that a surge in missile impacts is followed by a corresponding increase in patient arrivals. This synchronicity is evident during specific time frames, notably between 9–12 AM and 18 PM on October 7th, and again around 3 AM on October 8th. This surge in patients arriving at BUMCA's ED underscores the critical role the hospital plays in responding to the immediate aftermath of missile attacks. Figure 3, illustrating the Kaplan–Meier survival curve for patients admitted to the ED, portrays the temporal evolution of the probability of survival for this cohort. The perceptible descent in the curve signifies a diminishing likelihood of survival over time, while the gradual rate of decline in the Kaplan–Meier curve implies that a noteworthy proportion of these patients exhibit prolonged survival durations. Analysis of the 95% confidence intervals further elucidates the dynamics. Commencing with a narrow range of 0.73–0.99 at the onset, the intervals gradually widen, spanning 0.5–0.87 by the 8th hour, and further expanding to 0.16–0.55 at the 18th hour, remaining notably broad thereafter at 0.11–0.49 (Fig. 3). These widening intervals signify increased uncertainty regarding patient survival as time progresses.

## Discussion

The experience of BUMCA within the initial 24 h of the 2023 Hamas-Israel conflict offers valuable insights. While patient throughput met demand, opportunities to optimize trauma management in unstable settings were also identified [15,16]. By examining retrospective patient and hospital records from BUMCA's first 24 h, this case report aims to provide insights into the profile of trauma casualties treated as well as evaluate resuscitation protocols.

Understanding the specific challenges of rapidly receiving and stabilizing a mass influx of critically injured patients during ongoing hostilities can inform best practices for operating acute care services under fire. BUMCA’s experiences at the frontlines of the 2023 conflict provide a compelling case study for maintaining trauma services during armed conflict.

The most significant finding from our analysis concerned the outcome of patients who received emergent operative intervention. Reassuringly, there were no fatalities observed among the cases prioritized for urgent surgery, unequivocally highlighting the potentially life-saving impact of rapid access to the operating room (OR). This reinforced the long-standing principle in trauma care that during mass casualty incidents, patients with time-sensitive surgical needs should be triaged and treated above all others to maximize survival [17,18]. Delays in workup, transport, or provision of life-saving procedures for those with surgically treatable injuries likely would have directly contributed to preventable deaths [19]. While non-operative patients faced greater injury severity, decreasing OR capacity likely would have led to worse outcomes that defeating the goal of expanding throughput to save lives during the mass casualty event. Further analysis is still needed to fully understand relationships between clinical factors, OR management and mortality.

Our findings also suggest pathways for optimizing trauma surgical capability and reducing delays during mass casualty surges [19]. Examining issues that cause delay in processes such as triage transportation, and pre-operative care could identify targets for streamlining workflow [20]. For example, pre-establishing criteria for expediting certain injury types may decrease door-to-OR times. Enhancing communication and coordination between ED and OR staff could facilitate rapid patient transfers. Expanding surgical surge capacity through infrastructure improvements or load-balancing agreements with partner hospitals may help maintain OR access even under extreme demands. With further study of strategies employed at our and other trauma centers, protocols can be refined to maximize life-saving surgical interventions during catastrophic mass casualty events.

While the correlated pattern between missile impacts and patient arrivals at BUMCA underscoring the importance of surge preparation was an expected finding, further examination of the timing nuances revealed opportunities for improvement. Specifically, the delay of 30–60 min between impact and emergency department presentation warrants deeper analysis [21]. The variation in arrival times, with some within 30 min and others delayed up to 60 min, suggests potential points of bottleneck in the injury-to-care continuum that merit identification and addressing [22,23]. Factors such as distance from the attack, mechanisms of injury, pre-hospital triage protocols, and ambulance availability could partially explain the differential response windows. However, throughput challenges within the healthcare system itself may have also contributed, necessitating optimized mass casualty protocols [24,25].

While BUMCA successfully stabilized and transferred most patients within 24 h, the declining probability of survival over time and widening confidence intervals reflect the limitations imposed by injury severity, underscoring the value of earlier interventions. Pre-hospital care initiatives like frontline hemorrhage control protocols may help alter the clinical course of more critically injured patients upon arrival [26]. Equally, robust contingency plans are prudent to sustain trauma services for prolonged mass casualty incidents that test infrastructure and drain resources.

It is important to properly contextualize outcomes in relation to the spectrum and severity of injuries treated during this crisis. While staff exhibited exemplary skills and dedication, attributing survival solely to their acumen would neglect key clinical factors. The injuries presented ranged widely in their severity and likelihood of response to intervention. Even with expanded capacity and a highly coordinated response, some wounds sustained may have exceeded what could reasonably be compensated for, given resource constraints.

While surge capacity planning is well-established, analyzing the features of our response that navigated unique contextual challenges offers tangible lessons. The security volatility in this setting demanded innovative solutions beyond simply expanding facilities and staffing. Rather than generic flexibility recommendations, valuable insights emerged from how we streamlined care amid external restrictions. By pre-emptively positioning auxiliary teams near anticipated hotspots, incoming casualties received expedited triage and oversight. Leveraging secondary staging areas and deploying clinicians at points-of-injury addressed limitations to relying solely on infrastructure-based expansion within secure zones. Maintaining baseline care standards under continually changing demands required real-time coordination between main hospitals and alternate sites providing surrogate services. The ability to both supplement and substitute personnel at short notice according to evolving threats allowed pragmatic accommodation outside rigid facility-centric models. The integrated command structures used to optimize allocation of resources between fixed hospital facilities, mobile field clinics, and improvised treatment areas warrant further study. Examining how such coordinated response networks balanced surging demands across multiple locations could provide lessons for other organizations working to unify fragmented emergency medical systems during large-scale incidents. In settings with a predominance of male casualties engaged in frontline military or security duties, protocols and resources may be tailored accordingly. For example, anticipating common injury patterns like blast injuries from improvised devices allows the development of rapid diagnostics and targeted resuscitation protocols. Additionally, specialized surgical plans can be optimized based on the needs of these at-risk populations.

Patient survivability is significantly influenced by time to definitive care. As shown in our results, probability of survival based on the Kaplan–Meier curve diminished over 18 h from initial presentation. Widening confidence intervals indicate increased uncertainty about outcomes with prolonged time since injury. These findings emphasize the importance of minimizing delays, especially for those with time-sensitive needs. The concept of declining survival past the "golden hour" reinforces principles of prioritizing casualties likely to benefit most from prompt interventions [27]. Our triage protocols aimed to expedite care for those with limb threats, airway compromise or surgically treatable injuries.

Our model of prepositioning rapid response teams from partner trauma centers within the hospital helped streamline initial care without risks to external responders' safety. This decentralized approach supported maintaining the "golden hour" effect through minimal hand-off times. Lessons from coordinating regional assets during crises may guide other facilities. While one third of non-ambulatory admissions died, outcomes may have been worse without surging throughput [28].

Future efforts could explore optimizing injury severity stratification to better triage acuity and resource allocation during mass casualty events. Earlier identification of high-risk cohorts may help target interventions. Our experiences offer guiding principles for trauma systems operating in unstable areas to minimize delays and maximize survival potential.

On a practical level for mass casualty management, staffing teams with specialists experienced in treating combat trauma may help with triage and throughput. Establishing damage control procedures proven effective for penetrating injuries could also streamline the response. Ensuring adequate blood product reserves in ratios reflecting local wound epidemiology would support resuscitation efforts.

Maintaining optimal trauma services is made exceedingly difficult by threats to facilities and personnel safety from direct attacks. Hardening critical infrastructure through redundant power, shielding, and fireproofing helps sustain services under fire. Protecting staff through adequate personal protective equipment, timely sheltering drills and safety protocols is equally imperative. Healthcare neutrality efforts to immunize facilities from targeting could further support uninterrupted casualty care.

## Conclusion

This case study of BUMCA's response to the Hamas-Israel war offers valuable lessons for maintaining frontline emergency care during armed hostilities. By examining patient admission patterns, throughput measures and mortality trends over the initial 24 h of war, key challenges and opportunities for improvement have been identified. The experience underscores the need for hospitals near conflict zones to plan for recurring surges in mass casualties following spikes in violence. Dynamic surge capacity protocols and precautions to anticipate the first "wave" of critically injured patients are crucial. While BUMCA achieved timely overall throughput, targeting interventions for those with severe injuries early may help save more lives.

The predominance of military-aged males among casualties points to customized preparatory protocols addressing typical blast and multisystem trauma patterns. Concerted efforts must also sustain services through infrastructure hardening and staff safety given the constraints of operating under threat of direct attack.

While this analysis focused on operations within BUMCA, it is important to consider the hospital's role within the broader healthcare system. With processes to support flexible redirection of patients between facilities as needed, individual hospitals might have been relieved of some pressure during peak surges. Future studies could gain further insights by analyzing coordination and patient flows across the entire trauma network.

Overall, the findings provide a compelling case study for trauma management at the frontlines of armed conflict. By disseminating practical strategies gleaned from BUMCA's experience, other facilities could strengthen protocols optimizing frontline emergency care under hazardous conditions. Further research across diverse conflict settings may yield generalizable best practices. Ultimately, upholding healthcare neutrality to immunize facilities from targeting would best support uninterrupted delivery of life-saving care to combat casualties of war.

In summary, this retrospective analysis offers valuable lessons for operating hospitals near conflict zones. With optimized emergency preparedness drawing from such experiences, lives could be saved through improved coordination across the wider healthcare system.

## Data Availability

The analyzed data will be made available to requesting researchers upon a reasonable request.
